# Adherence of French GPs to Chronic Neuropathic Pain Clinical Guidelines: Results of a Cross-Sectional, Randomized, “e” Case-Vignette Survey

**DOI:** 10.1371/journal.pone.0093855

**Published:** 2014-04-18

**Authors:** Valéria Martinez, Nadine Attal, Bertrand Vanzo, Eric Vicaut, Jean Michel Gautier, Didier Bouhassira, Michel Lantéri-Minet

**Affiliations:** 1 Anesthésiologie-Réanimation, Hôpital Raymond-Poincaré, Garches, France; 2 INSERM U-987, Centre d'Evaluation et de Traitement de la Douleur, CHU Ambroise Paré, Boulogne-Billancourt, France; 3 Université Versailles-Saint-Quentin, Versailles, France; 4 General Practitioner, Athis Mons, France; 5 Unité de Recherche Clinique - Hôpital Fernand Widal, Paris, France; 6 Réseau InterCLUD Languedoc Roussillon, CHRU Montpellier, Montpellier, France; 7 CHU de Nice, Centre d'Evaluation et Traitement de la Douleur, Nice, France; 8 INSERM/UdA, U1107, Neuro-Dol, Université de Clermont-Ferrand, Clermont-Ferrand, France; University of Louisville, United States of America

## Abstract

**Background and aims:**

The French Pain Society published guidelines for neuropathic pain management in 2010. Our aim was to evaluate the compliance of GPs with these guidelines three years later.

**Methods:**

We used “e” case vignette methodology for this non interventional study. A national panel of randomly selected GPs was included. We used eight “e” case-vignettes relating to chronic pain, differing in terms of the type of pain (neuropathic/non neuropathic), etiology (cancer, postoperative pain, low back pain with or without radicular pain, diabetes) and symptoms. GPs received two randomly selected consecutive “e” case vignettes (with/without neuropathic pain). We analyzed their ability to recognize neuropathic pain and to prescribe appropriate first-line treatment.

**Results:**

From the 1265 GPs in the database, we recruited 443 (35.0%), 334 of whom logged onto the web site (26.4%) and 319 (25.2%) of whom completed the survey. Among these GPs, 170 (53.3%) were aware of the guidelines, 136 (42.6%) were able to follow them, and 110 (34.5%) used the DN4 diagnostic tool. Sensitivity for neuropathic pain recognition was 87.8% (CI: 84.2%; 91.4%). However, postoperative neuropathic pain was less well diagnosed (77.9%; CI: 69.6%; 86.2%) than diabetic pain (95.2%; CI: 90.0%; 100.0%), cancer pain (90.6%; CI: 83.5%; 97.8%) and typical radicular pain (90.7%; CI: 84.9%; 96.5%). When neuropathic pain was correctly recognized, the likelihood of appropriate first-line treatment prescription was 90.6% (CI: 87.4%; 93.8%). The treatments proposed were pregabaline (71.8%), gabapentine (43.9%), amiptriptylline (23.2%) and duloxetine (18.2%). However, ibuprofen (11%), acetaminophen-codeine (29.5%) and clonazepam (10%) were still prescribed.

**Conclusions:**

The compliance of GPs with clinical practice guidelines appeared to be satisfactory, but differed between etiologies.

## Introduction

Neuropathic pain constitutes a significant burden for society in terms of impaired quality of life, comorbidities and cost [1]–[3]. Classical causes of neuropathic pain include diabetes, shingles, spinal cord injury, stroke, multiple sclerosis, cancer and HIV infection, but also more common conditions, such as radicular pain related to radiculopathy, and traumatic or postsurgical nerve injuries [4].

Several large epidemiological surveys have highlighted the under-treatment of neuropathic pain in France in either the general population [5] or in specialist settings [6]. Screening tools have been developed to increase the awareness and recognition of neuropathic pain, particularly by non-specialists [7]–[9]. The use of these tools may also contribute to reduce false positive diagnoses, which are probably also common in clinical practice.

Evidence-based recommendations for the assessment and management of neuropathic pain have also been developed in recent years [10]–[13]. In France, the French Pain Society (*Société Française d'Etude et Traitement de la Douleur*/SFETD), in particular, has proposed and disseminated evidence-based recommendations targeting all health professionals, with the aim of facilitating neuropathic pain recognition and management in the ambulatory care setting [14]. These recommendations emphasize the importance of screening tools (particularly the DN4, which was developed in France [7]) as a first step in the diagnosis of neuropathic pain, and propose first- and second-line drug treatments for neuropathic pain, regardless of its etiology, akin to European or international recommendations.

However, very few studies have investigated the real-life impact of evidence-based recommendations on physicians' practices. In the USA, a study by Dworkin and colleagues [15] suggested that the drug treatment of post-herpetic neuralgia by primary care physicians was roughly consistent with the US recommendations issued some years before. However, this study was retrospective and restricted to post-herpetic neuralgia, a condition that is easier for non-specialists to diagnose than many other neurological pain conditions.

Our aim in this study was to describe chronic neuropathic pain management practices among general practitioners (GPs), focusing on the criteria used in decision-making processes and compliance with current French recommendations. We used “case vignettes”, a valid and reliable method that is gaining widespread acceptance for quality-of-care assessments in current clinical practice [16]–[20]. This method provides an effective evaluation of the behavior of physicians in the setting of diagnosis or treatment decisions, and of their compliance with recommendations. It therefore appeared an appropriate method in the context of the objectives of this study.

## Materials and Methods

### Ethics statement

This study was conducted in accordance with French regulatory requirements. The protocol and all administrative documents, including the financial agreement, with investigators paid for their participation, were approved by the National Medical Council (*Conseil National de l'Ordre des Médecins*; CNOM). The database was declared to the National Data Protection Authority (*Commission Nationale de l'Informatique et des Libertés*; CNIL). Submission to an ethics committee was not required under French law.

### Selection of participants

**Figure 1:**
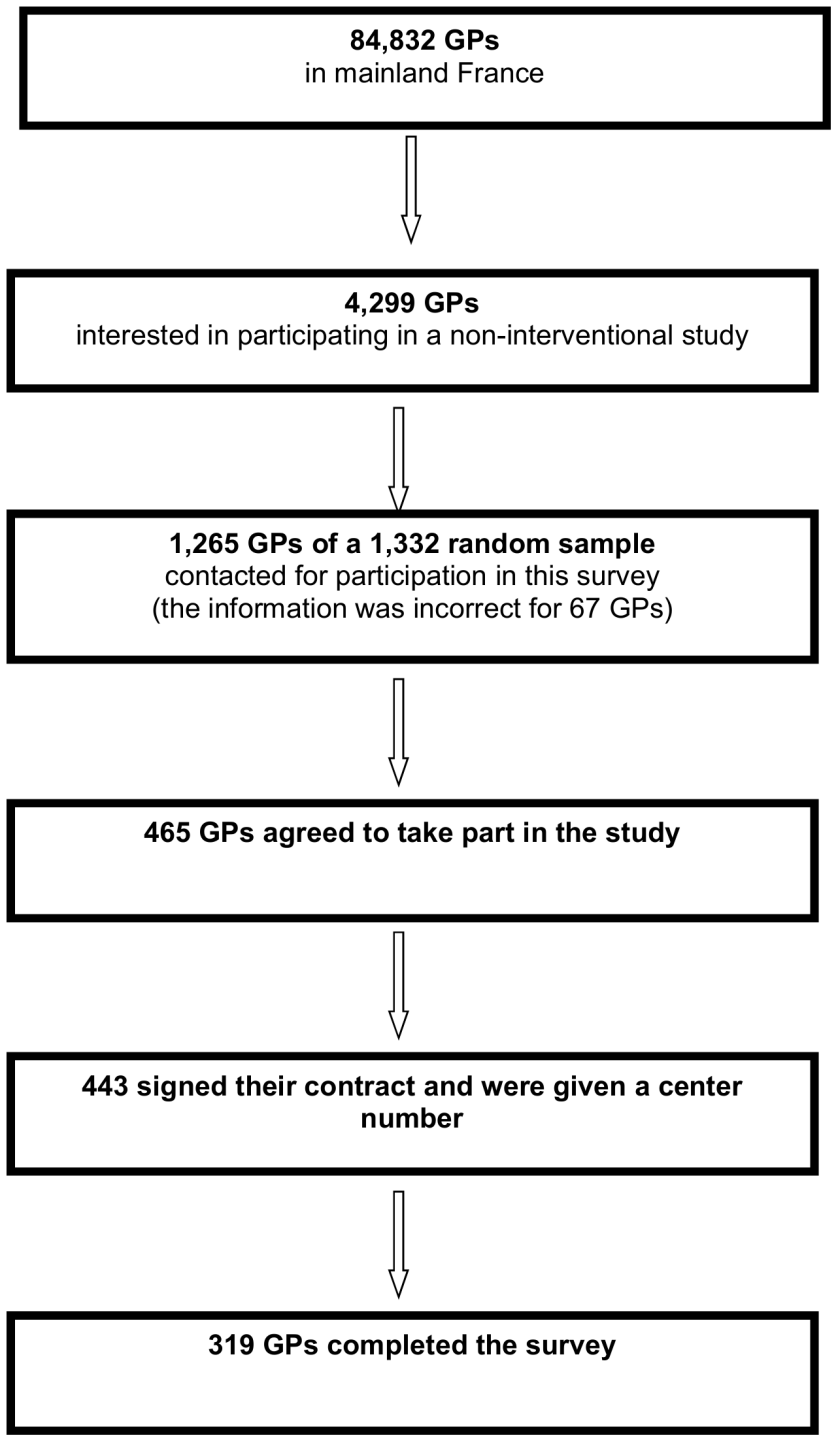
Flow chart of the study.

We calculated that a sample of 300 GPs would be required to estimate any percentage with a maximum 95% CI of ±6.5%, taking into account a design effect related to the non-independence of observations made by the same physician. The rate of participation in e-CRF studies is generally about 30%, with 75% of participants being highly active. We therefore decided to select a representative random sample of 1,332 GPs for this study. To this end, we sent a questionnaire, by mail, to the 84,832 GPs practicing in mainland France (listed in the CEGEDIM database), on January 2^nd^, 2012, asking them whether they would be interested in participating in a non-interventional study. We selected a random sample of 1,332 physicians from the 4,299 GPs who expressed interest in participating in this study between January and July 2012. Contact information was incorrect for 67 GPs, so 1,265 GPs were finally included in our database. In October 2012, we sent these GPs a proposal for participation in this survey. Any physician who did not respond to this mailing was contacted by telephone two weeks later, and attempts at contact were continued until the planned number of participants was reached. In total, 465 GPs agreed to take part in this survey, of whom 443 signed a contract for participation and were issued with a center number. The flow chart for GP selection is summarized in Figure 1.

### Construction of case-vignettes

Case vignettes were developed by a multidisciplinary panel of experts (authors of this paper) in the field of neuropathic pain, practicing as GPs, clinicians or nurses in neurology or anesthesiology. The description of clinical cases was based on epidemiological and descriptive data characterizing neuropathic pain [21]. Each clinical case was designed to be realistic and concrete, matching as closely as possible the cases observed in clinical practice. We focused on four etiologies of peripheral neuropathic pain frequently encountered in general medical practice: painful diabetic polyneuropathy, cancer chemotherapy-induced peripheral neuropathy [22], typical radicular pain [23] and post-operative neuropathic pain [24]–[26]. In assessments of performance for the identification of neuropathic pain, we considered two types of chronic pain for each etiology: nociceptive chronic pain and peripheral neuropathic pain. Based on these conditions, we constructed eight case-vignettes, each with a different scenario. The eight case-vignettes are given in File S1.

The scenario needed to be simple, brief (length <15 lines-200 words), with no potential pitfalls. Each case-vignette was constructed in the same way: age, sex, patient's history, chronic pain history, reason for consultation, clinical symptoms, data from clinical examination, with or without results from additional investigations.

Scenarios differed in terms of both symptoms (e.g. burning pain *vs* aching pain) and elements of clinical examination (e.g. allodynia *vs* pain triggered by joint mobilization). The symptoms and clinical examination findings leading to the diagnosis of neuropathic pain were chosen from among the most discriminative, although none of them is specific [7], [11], [27] and were consistent with those used in validated screening tools, such as the DN4 (*Douleur neuropathique en 4 questions*) [7]. Clinical cases of neuropathic pain included at least six discriminative neuropathic elements, whereas non-neuropathic cases presented fewer than two neuropathic characteristics (see cases n°7 and 8 in File S1).

Non-discriminative clinical elements of neuropathic pain were systematically included in the description of all clinical cases: high pain intensity on a numerical rating scale (>7/10) and comorbid conditions (anxiety, depression).

### Construction of the case-vignette questionnaire

The proposed questions and their corresponding items dealt with the different elements for the diagnosis and management of neuropathic pain set out in SFETD recommendations (see Key points in File S1). The construction of this closed-ended questionnaire superimposed over the recommendations made it possible to carry out a relevant assessment of knowledge and facilitated the analysis of responses. The number of questions and items was the same for each case. The knowledge of the GPs was assessed through four multiple-choice questions: i) diagnostic elements, four items, ii) elements from the patient's clinical history guiding diagnosis, four items, iii) elements from clinical examination guiding diagnosis, three items, iv) drugs proposed for first-line treatment, seven items.

The case-vignette questionnaire is shown in in File S1. The list of first-line drugs includes duloxetine, which is authorized only for the treatment of painful diabetic peripheral neuropathy in France.

### Procedure

Eight case-vignettes were constructed, corresponding to four neuropathic cases and four non-neuropathic cases (Table 1). By combining these vignettes two-by-two (one neuropathic case and one non-neuropathic case), we obtained 12 possible combinations, which we tested on 20 GPs, to evaluate their comprehensibility. This testing step identified two combinations as too similar to each other (diabetic polyneuropathy and cancer chemotherapy-induced polyneuropathy), and these combinations were therefore removed. Each GP had to provide a response for a set of two case-vignettes randomly selected from the 10 remaining combinations. GPs were blind to the mode of case distribution.

**Table 1:** The eight case-vignettes.

	Neuropathic pain	Non-neuropathic pain
Diabetes	Case 1	Case 2
Cancer	Case 3	Case 4
Low back pain	Case 5	Case 6
Postoperative pain	Case 7	Case 8

The case-vignettes were stored in a database on a dedicated server for this survey (a web-based application). GPs could access their two assigned case-vignettes at any time, *via* a personal online account protected by a specific and confidential login and password assigned to them after validation of the financial agreement. Once all fields of the case-vignette questionnaire had been completed, the questionnaire was saved automatically on the administrator's account, preventing any further modification.

In the event of an incorrect diagnosis after the response to the first three questions, GPs were given the correct diagnosis so that they could answer the question relating to therapeutic strategy with the correct diagnosis in mind.

Before completing the case-vignette questionnaires, the participants provided the following information: their age, sex, duration of practice, practice in an urban/rural area, number of chronic pain patients seen per week (and the percentage of these patients with neuropathic pain). After validation of the responses, they were also asked the following questions: *“Are you aware of the SFETD recommendations? Do you implement them? Are you aware of the DN4 tool* (Douleur Neuropathique en 4 questions)? *Do you use it?”*

### Statistical analysis

For continuous variables, we determined the mean and standard deviation. For categorical variables, the number and percentage of subjects in each category are summarized. We used Student's *t* test to assess differences for continuous variables. Chi^2^ tests of association were used to test for sequence order differences for categorical variables. The threshold for significance was set at *p* = 0.05.

We determined the percentage of cases correctly diagnosed, with its 95% confidence interval (CI). A similar analysis was performed for each of the eight case-vignettes, according to the type of pain (neuropathic or non-neuropathic) and the underlying disease. The percentages of GPs making 0, 1 and 2 corrected diagnoses were determined.

The elements of the patient's history and clinical examination used to reach the diagnosis were described separately for each of the eight case-vignettes, according to the type of pain (neuropathic or non-neuropathic) and the disease, and for the following subgroups: correct diagnosis/misdiagnosis, knowledge of recommendations (YES/NO) and their implementation (YES/NO), knowledge of the DN4 tool (YES/NO) and its use (YES/NO). The results obtained were compared with the expected responses.

Prescriptions of analgesic treatments were described separately for each of the eight case-vignettes and according to: the type of pain (neuropathic or non-neuropathic), knowledge of the recommendations (YES/NO) and their implementation (YES/NO).

We aimed to evaluate compliance with SFETD guidelines. Thus, for each question on the case-vignette questionnaire, we analyzed the number of correct answers and the number of answers containing at least one element of the correct answer, without any element wrongly ticked.

Analyses were performed with SAS version 9.3 (SAS Institute, Cary, NC).

## Results

In total, 443 GPs signed a contract and received a center number, but only the 319 duly completing the e-CRF were included in the analysis. We thus evaluated responses for a total of 638 case-vignettes.

### Characteristics of connections

An analysis of connection times showed that the participants remained connected for a median of 17 minutes and that the minimum connection time was four minutes. A few extreme values were obtained, probably due to participants forgetting to sign out.

### Characteristics of the participants

The demographic features of the 319 GPs completing the questionnaires for both the clinical cases assigned to them are reported in Table 2.

**Table 2:** GPs' characteristics.

	Statistics	*N* = 319
Sex		
*Male*	*n(%)*	254 (79.6%)
*Female*	*n(%)*	65 (20.4%)
Pattern of medical practice		
*Alone*	*n(%)*	125 (40.1%)
*Group practice*	*n(%)*	187 (59.9%)
	*Missing (n - %)*	7 (2.2%)
Urban/Rural		
*Rural*	*n(%)*	191 (59.9%)
*Urban*	*n(%)*	128 (40.1%)
Age (years)	*MeanSD*	52.3
	*Median*	8.4
	*Q1;Q3*	53.0
	*[Min; Max]*	[47.0;59.0]
		[31.0;69.0]
Duration of practice		
*<10 years*	*n(%)*	33 (10.3%)
*> = 10 years*	*n(%)*	286 (89.7%)
Number of chronic pain patients per month	*Mean*	52.8
	*SD*	46.7
	*Median*	45.0
	*Q1;Q3*	[20.0;70.0]
	*[Min; Max]*	[2.0;400.0]
Number of chronic pain patients with neuropathic pain per month	*Mean*	8.6
	*SD*	10.8
	*Median*	5.0
	*Q1;Q3*	[2.5;10.0]
	*[Min; Max]*	[0.1;100.0]

SD, standard deviation; Q1–Q3, first and third quartiles; min, minimum; max, maximum.

This sample of physicians was representative of the general population of GPs in terms of their nationwide distribution (with a slight underrepresentation of Ile-de-France) and mean age. Men were overrepresented (79.6% *vs* the expected 59%, based on GP numbers) as frequently reported in studies of this type. Representativeness was assessed on the basis of the French atlas of medical demography (Atlas National CNOM 2012).

Of the 319 GPs completing the questionnaires for both case-vignettes, 53.3% (170/319) were aware of guidelines and 42.6% (136/319) said that they implemented them. The principal reason for not following the recommendations was insufficient knowledge of these recommendations. For the DN4 diagnostic tool, 60.8% of the GPs (194/319) stated that they were aware of it and 34.5% (110/319) reported using it. The main reason for not using this tool was a lack of memorized knowledge.

### Evaluation of case-vignettes: diagnosis and therapeutic strategy

#### Global results

**Figure 2:**
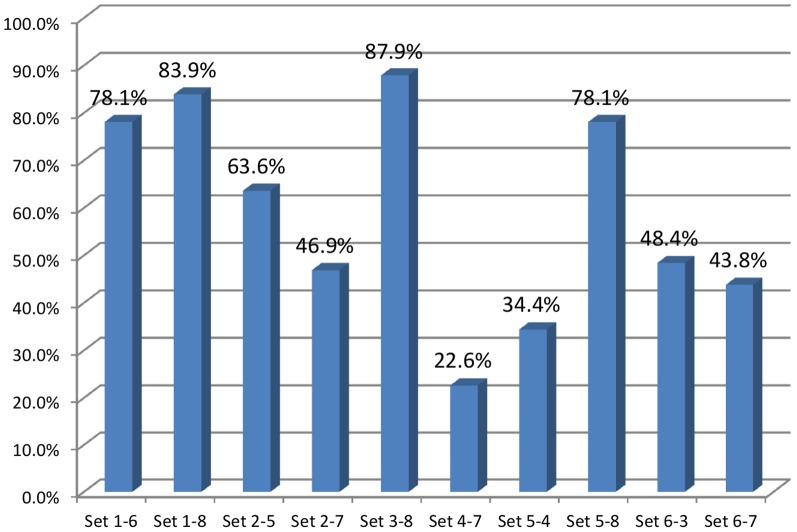
Proportion of investigators (*N* = 319) with 2 correct diagnoses.

***Of the 319 GPs***, 58.9% made the correct diagnosis for both the allocated case vignettes. The proportion of GPs giving two correct diagnoses did not between the sexes (58.7% men *vs* 60.0% women; *p* = 0.845) or between types of practice are (56.0% rural *vs* 63.3% urban; *p* = 0.434). The number of accurate diagnoses did not depend on whether the doctors were aware of or implemented the recommendations; similar results were obtained concerning knowledge and use of the DN4 tool. Several case combinations were identified less well than others. The proportions of GPs giving the correct diagnosis for both case vignettes was lowest (Figure 2) when one of the allocated vignettes was a case of cancer with non-neuropathic pain (case no. 4, with case no. 7: 22.6%, or with case no. 5: 34.4%). Postoperative neuropathic pain (case no. 7) also appeared to be difficult to identify, as shown for its combination with case no. 6 (43.8%) or case no. 2 (46.9%).

**Figure 3:**
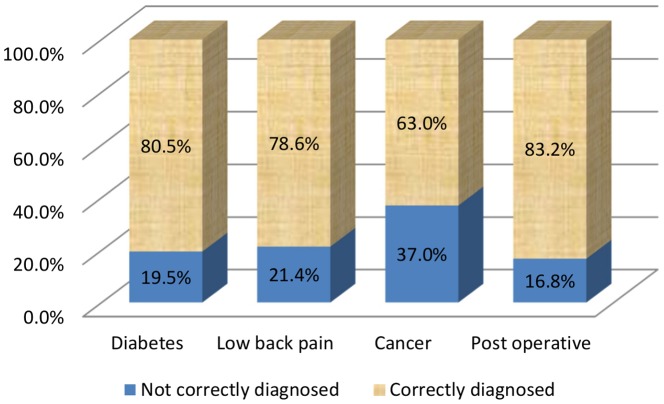
Frequency of correct/incorrect diagnoses by etiology (*N* = 638).

***For the 638 vignettes examined***, the correct diagnosis was made in 77.3% of cases. The percentage of correct diagnoses differed considerably between etiologies and was lowest for cancer cases (p = 0.0002; Figure 3).

**Figure 4:**
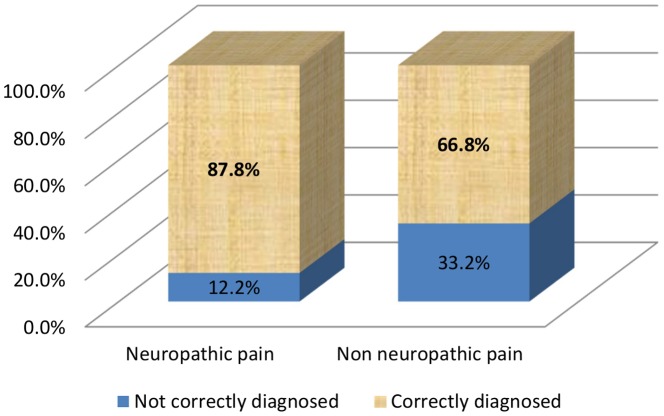
Frequency of correct/incorrect diagnoses by type of pain (*N* = 638).

**Figure 5:**
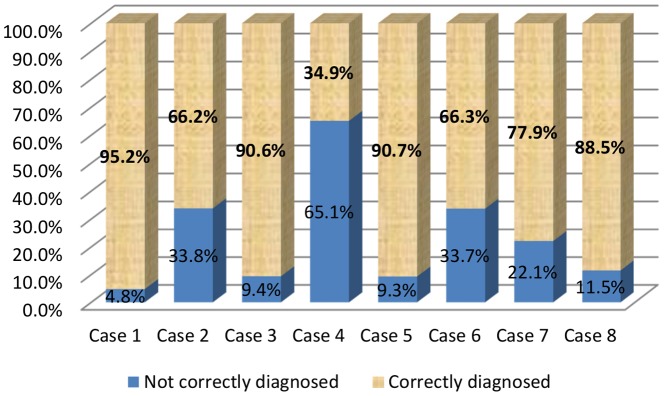
Frequency of correct/incorrect diagnoses for each case-vignette (*N* = 638).

***Neuropathic pain*** was well diagnosed in 87.8% (95%CI: 84.2%; 91.4%) of cases (Figure 4), but the frequency of correct diagnosis differed between etiologies (Figure 5). The probability of correct diagnosis seemed to be lower for postoperative neuropathic pain (case no. 7) (77.9%; 95%CI [69.6%; 86.2%]) than for other etiologies (92.0%; 95%CI [88.4%; 95.5%]). Thus, misdiagnosis was 2.8 times more frequent for postoperative neuropathic pain than for other etiologies (22.1% *vs* 8.0%; *p* = 0.0005).

Our analysis of the criteria on which GPs based their diagnosis of neuropathic pain indicated that all items from the patient's history and all items from the clinical examination characterizing this type of pain were recognized in 53.9% and 51.8% of cases, respectively (Table 3). For the 280 GPs making the correct diagnosis, 112 (40%) had ticked at least four correct answers among the seven items proposed; this percentage was significantly lower among the GPs giving the wrong diagnosis (9 of 39 GPs i.e. 23.1%; *p* = 0.041). A case-by-case analysis of the results highlighted differences in the identification of key diagnostic elements as a function of etiology. Indeed, for diabetic neuropathic pain (case no. 1), diagnosis was based mostly on the patient's history rather than clinical examination, whereas, for postoperative neuropathic pain (case no. 7) the key diagnostic elements were more easily identified from clinical examination than from the patient's history. These results are summarized in Table 4.

**Table 3:** Key elements for the correct diagnosis of neuropathic pain.

Etiology	Elements from history-exact response rate-	Elements from clinical examination-exact response rate-	p(Mc Nemar)
Diabetes	66.7% (40/60)	35.0% (21/60)	0.0009
Cancer	55.2% (32/58)	56.9% (33/58)	0.8273
Low back pain	54.5% (48/88)	46.6% (41/88)	0.2967
Postoperative pain	41.9% (31/74)	67.6% (50/74)	0.0038
All cases of neuropathic pain	53.9% (151/280)	51.8% (145/280)	0.6146

**Table 4:** Key elements leading to correct diagnosis and choice of therapeutic strategy for each of the 8 case-vignettes.

	Case-vignette							
	1	2	3	4	5	6	7	8
	*n* = 63	*n* = 65	*n* = 64	*n* = 63	*n* = 97	*n* = 95	*n* = 95	*n* = 96
ELEMENTS FROM HISTORY								
Exact answer* (%)	66.7%	46.5%	55.2%	45.5%	54.5%	42.9%	41.9%	60.0%
Correct answer** (%)	71.7%	72.1%	58.6%	45.5%	56.8%	55.6%	45.9%	64.7%
ELEMENTS FROM CLINICAL EXAMINATION								
Exact answer* (%)	35.0%	97.7%	56.9%	81.8%	46.6%	61.9%	67.6%	49.4%
Correct answer (%)	100%***	97.7%	70.7%	81.8%	100%***	61.9%	100%***	77.6%
CORRECT PRESCRIPTIONS								
All first-line drugs proposed	7.9%	41.5%	3.1%	30.0%	5.2%	36.8%	6.3%	37.5%
At least one first-line drug proposed	73.0%	70.8%	51.6%	65.1%	44.3%	62.1%	51.6%	59.4%

* an answer strictly corresponding to the expected answer (the exact answer required all items to be ticked).

** an answer with at least one element of the exact answer, without any element wrongly ticked.

We found that 53.6% GPs faced with a case of neuropathic pain of any etiology prescribed at least one first-line treatment recommended for this type of pain. Significant differences in prescriptions were observed as a function of etiology. Indeed, a first-line treatment was prescribed for 73.0% of diabetes cases but only 48.8% cases of neuropathic pain of other etiologies (*p* = 0.0006). Only 4.7% of the GPs identified all the drugs that could be used as first-line treatments for neuropathic pain. A significant disparity in drug prescriptions was observed, with pregabalin prescribed in 71.8% of cases, gabapentin in 43.9% and amitriptyline in 23.2%. The orders of the prescription rates for these three drugs did not depend on etiology. However, for diabetic polyneuropathy, duloxetine replaced amitriptyline as the third most frequently prescribed drug. The questions relating to therapeutic approaches also revealed that many doctors would prescribe inappropriate drugs, such as acetaminophen/codeine, ibuprofen and clonazepam, which were prescribed in 29.5%, 11% and 10% of cases, respectively. Data on drug prescriptions in neuropathic pain are presented in Table 4 and Table 5.

**Table 5:** Correct prescription rates for neuropathic pain.

Drugs	*N* = 319
Pregabalin/amitryptyline/gabapentin*	15 (4.7%)
Pregabalin	229 (71.8%)
Amitryptyline	74 (23.2%)
Gabapentin	140 (43.9%)
At least one recommended drug **	171 (53.6%)

*all three first-line recommended drugs.

** and no incorrect treatment.

***Non-neuropathic pain*** was well diagnosed in 66.8% of cases. Postoperative pain (case no. 8) was the easiest to identify and cancer pain (case no. 4) proved to be the most difficult. An overdiagnosis of neuropathic pain was observed, with 33.2% of the 319 cases of non-neuropathic pain incorrectly diagnosed as neuropathic pain and a clear predominance of incorrect diagnoses for cancer pain (65.1%). These results are presented in Figures 4 and 5.

Correct diagnosis, with all the correct items ticked for history or clinical examination, was observed in 50.7% and 66.2% of cases, respectively. Other than for postoperative pain, the key elements for a correct diagnosis were more frequently obtained from clinical examination than from history (Table 6).

**Table 6:** Key elements for the correct diagnosis of non-neuropathic pain.

Etiology	Elements from history-exact response rate-	Elements from clinical examination-exact response rate-	p(Mc Nemar)
Diabetes	46.5% (20/43)	97.7% (42/43)	<0.0001
Cancer	45.5% (10/22)	81.8% (18/22)	0.0047
Low back pain	42.9% (27/63)	61.9% (39/63)	0.0233
Postoperative pain	60.0% (51/85)	49.4% (42/85)	0.1797
All cases of neuropathic pain	50.7% (108/213)	66.2% (145/213)	0.0011

In terms of therapeutic strategy, regardless of the etiology of non-neuropathic pain, about one third of GPs (36.7%) prescribed both appropriate drugs — ibuprofen and acetaminophen/codeine — and about two thirds (63.6%) prescribed at least one of these two drugs. Correct prescription rates were similar whether or not the recommendations were applied (Table 7).

**Table 7:** Correct prescription rates for non-neuropathic pain.

Drugs	*N* = 319
Ibuprofen/acetaminophen-codeine*	117 (36.7%)
Ibuprofen	200 (62.7%)
Acetaminophen-codeine	248 (77.7%)
At least one recommended drug **	203 (63.6%)

* the two first-line recommended drugs.

** and no incorrect treatment.

For both neuropathic and non-neuropathic pain, knowledge and implementation of the recommendations, and knowledge and use of the DN4 tool, did not appear to affect the likelihood of recognizing key elements from the patient's history or clinical examination for accurate diagnosis.

#### Case-by-case results

***Painful diabetic polyneuropathy*** (case no. 1) was well diagnosed in most cases (95.2%), with 66.7% and 35.0% exact answers for the patient's history and clinical examination, respectively. In terms of therapeutic strategy, the complete list of recommended first-line treatments was rarely given (7.9%), but one of the drugs from this list was proposed in about three in four cases (73.0%). The drug most frequently proposed was pregabalin (73.0% of cases).

***Non-neuropathic pain in diabetic patients*** (case no. 2) was correctly diagnosed in 66.2% of cases, with 46.5% and 97.7% exact answers for the patient's history and clinical examination, respectively. If we considered cases with only one element of the exact answer but with no incorrectly ticked elements, these percentages were 72.1% and 97.7%, respectively. In cases of incorrect diagnosis, no exact answer was obtained for either history or clinical examination. For therapeutic strategy, the two recommended first-line drugs (ibuprofen and acetaminophen/codeine) were proposed together in less than half the cases (41.5%), but one of these drugs was cited in 70.8% of cases. Acetaminophen/codeine was the most frequently proposed drug (80.0% of cases).

***Cancer chemotherapy-induced peripheral neuropathy*** (case no. 3) was well diagnosed in 90.6% of cases, with 55.2% and 56.9% exact answers for the patient's history and clinical examination, respectively. If we considered answers with at least one correct element, these percentages were 58.6% and 70.7%, respectively. For therapeutic management, very few GPs (3.1%) identified all three recommended first-line drugs, but half (51.6%) proposed one of these drugs. The drug most frequently identified was pregabalin (75.0% of cases).

***Non-neuropathic pain in cancer*** (case no. 4) was diagnosed in only 34.9% of cases, with 45.5% and 81.8% exact answers for the patient's history and clinical examination, respectively. Similar results were obtained if we considered responses with at least one correct element. In cases of incorrect diagnosis, no exact answer was obtained for either the patient's history or for clinical examination. The two recommended first-line drugs (ibuprofen and acetaminophen/codeine) were both proposed in 30.2% of cases, but at least one of these two drugs was cited in 65.1% of cases. Acetaminophen/codeine was the most frequently proposed drug (79.4% of cases).

***Typical radicular pain*** (case no. 5) was correctly identified in 90.7% of cases, with 54.5% and 46.6% exact answers for the patient's history and clinical examination, respectively. Very few GPs (5.2%) identified all three recommended first-line drugs, and only 44.3% proposed one of these drugs. The most frequently cited drug was pregabalin (64.9% of cases).

***Non-neuropathic low back pain*** (case no. 6) was correctly diagnosed in 66.3% of cases, with 42.9% and 61.9% exact answers for the patient's history and clinical examination, respectively. If we considered answers with at least one correct element, these percentages were 55.6% and 61.9%, respectively. In cases of incorrect diagnosis, exact answers for history and clinical examination were observed in only 9.4% and 3.1% of cases, respectively. The two recommended first-line drugs (ibuprofen and acetaminophen/codeine) were both proposed by 36.8% of GPs, and one of these drugs was proposed by 62.1% of GPs. Acetaminophen/codeine was the most frequently proposed drug (83.2% of cases).

***Postoperative neuropathic pain*** (case no. 7) was well diagnosed in 77.9% of cases, with 41.9% and 67.6% exact answers for the patient's history and clinical examination, respectively. In cases of incorrect diagnosis, exact answers from the patient's history and clinical examination were obtained in 33.3% and 28.6% of cases, respectively. For therapeutic strategy, all three recommended first line treatments was rarely ticked together (6.3%), but one of these drugs was proposed in about half the cases (51.6%). The most frequently cited drug was pregabalin (75.8% of cases).

***Postoperative non-neuropathic pain*** (case no. 8) was diagnosed in 88.5% of cases, with 60.0% and 49.4% exact answers for the patient's history and clinical examination, respectively. If we considered answers with at least one correct element, these percentages were 64.7% and 77.6%, respectively. In cases of wrong diagnosis, no exact answer was given for either history or clinical examination. The two recommended first-line drugs (ibuprofen and acetaminophen/codeine) were both proposed by 37.5% of GPs, and one of these drugs was proposed by 59.4% of GPs.

## Discussion

This study is the first to address the issue of the compliance of French general practitioners with current recommendations for the diagnosis and first-line treatment of neuropathic pain [14]. Three years after the publication of French recommendations on neuropathic pain, this study found that only 58.9% of GPs made the correct diagnosis for both the allocated case vignettes. Neuropathic pain was well diagnosed in 87.8% of cases, but only 53.6% of GPs proposed an appropriate first-line treatment. Based on these figures, less than one in two patients (47.1%) consulting for neuropathic pain would receive appropriate treatment.

### Diagnosis of neuropathic pain

Accurate diagnosis is the crucial first step toward the successful management of neuropathic pain. We were surprised by the high percentage of correct diagnoses of neuropathic pain obtained in this study (87.8%), given the difficulties encountered in the recognition of this type of pain real life [28], [29]. However, the clinical cases submitted to the GPs in this study were simple and somewhat caricatured. Our cases of neuropathic pain included at least seven characteristic features of such pain, whereas four elements are sufficient for the diagnosis of NP with the DN4 tool [7]. Moreover, it has been shown that the use of closed-ended questionnaires with cued items leads to an overestimate of the performance of physicians [30]. The sponsorship of this study by a laboratory heavily involved in research on neuropathic pain may also have resulted in a higher proportion of GPs making the correct diagnosis for neuropathic pain. Thus, although the overall figures appear to be highly satisfactory, further analyses of diagnostic failures can provide us with useful information. Indeed, this detailed analysis identified certain diagnostic difficulties in particular conditions.

Firstly, the large range of answers obtained, depending on the set of two case-vignettes assigned to the GPs, suggested that diagnosis was not always easy. Some cases, such as painful diabetic polyneuropathy, seemed to be much better diagnosed than others, such as postoperative neuropathic pain. We also observed an overdiagnosis of neuropathic pain in cancer patients. Only one in five GPs recognized both non-neuropathic cancer pain and postoperative neuropathic pain. The overdiagnosis of neuropathic pain in some etiologies and its underdiagnosis in others may reflect GPs continuing to think that the nature of pain depends on either its context or its etiology. Parsons *et al.* recently showed that mean time from the onset of postoperative neuropathic pain symptoms to diagnosis was 9.7 months [29]. Indeed, postoperative pain had long been considered purely, until the widespread recognition about 10 years ago of the neuropathic origin of some postoperative pains [31]. By contrast, diabetic neuropathy has been the subject of extensive academic, scientific communication and marketing, potentially accounting for the high frequency of correct diagnosis (92.2%). GPs overdiagnosed neuropathic pain in cancer patients, probably because they were influenced by the background of previous chemotherapy and the presence of two neuropathic elements (e.g. tingling and hypoesthesia) without taking into account clinical presentation (e.g. location of pain away from nerve damage symptoms).

Overall, these results suggest that limited use is made of the findings of clinical examinations in the diagnosis of neuropathic pain. This hypothesis was confirmed by the analysis of answers relating to the elements of the patient's history and clinical examination leading to the diagnosis of neuropathic pain, which provided us with new insight into the decision-making processes of physicians. Indeed, our results indicate that the elements used to arrive at the correct diagnosis differed between etiologies. In diabetic patients, pain descriptors were used for the identification of neuropathic pain, whereas, in postoperative pain, correct diagnoses were based largely on clinical examination. This is a key point, highlighting the essential nature of clinical examination for the diagnosis of neuropathic pain in general practice. This finding closes the debate about whether clinical examinations should be counted among the screening tools helping non-specialists to identify patients with possible neuropathic pain.

### Therapeutic management

The management of patients with chronic neuropathic pain is challenging, despite attempts to develop a more rational therapeutic approach [32]. In our study, only one in two GPs proposed at least one of the recommended first-line treatments when faced with a case of confirmed neuropathic pain. This poor result applied to neuropathic pain of all etiologies. Indeed, when multiplied with the probability of prescription of one appropriate first-line treatment, only 69.5%, 46.7%, 40.2% and 40.2% of patients with diabetes, cancer, radicular pain and postoperative pain, respectively, would receive appropriate medical care. These low rates highlight the large proportion of patients that would not have been correctly treated despite the caricatured description of neuropathic pain in the vignettes, its high intensity (7/10 on VAS) and association with sleep disorders and anxiety in all the case-vignettes assigned to the GPs. These findings suggest that the corresponding figures may be even worse in “real life”. As for diagnosis, we observed differences according to etiology with neuropathic pain in diabetic patients much better managed than that in patients presenting with low back pain or postoperative pain.

Another key finding of our results is the lack of knowledge of evidence-based therapy, with less than 5% of GPs being able to list all the first-line drugs recommended for the treatment of neuropathic pain. This suggests that physicians have a very superficial knowledge of recommendations, such that patients with contraindications or treatment failure might not necessarily receive appropriate treatment. It is probably easier for GPs to remember one drug per disease, even if further inquiries must subsequently be made, rather than remembering algorithms of various complexities. Pregabalin was by far the most frequently cited drug, regardless of etiology, followed by gabapentin and amitriptyline. This finding may be accounted for by the risk-benefit profile of pregabalin, which is better tolerated and requires fewer precautions for use than amitriptyline [14]. Finally, this study revealed the persistence of non-recommended drug prescriptions, such as acetaminophen/codeine, ibuprofen or clonazepam. Clonazepam, which belongs to the benzodiazepine class, has been misused and abused and has been subject to prescription limitations imposed by the French authorities since January 2012 [33]. The other drugs in this non-recommended list are very often wrongly prescribed, to one inthree patients for ibuprofen and one in five patients for acetaminophen/codeine. Nevertheless, non-recommended drug prescriptions were less frequent than reported in a recent study investigating the treatment of neuropathic pain in the UK general population [34]. An opioid, or a combination of opioid and non-opioid analgesics, was prescribed as a first-line treatment for 25.4% of patients with diabetic neuropathy and 64.0% of patients with neuropathic back pain. By contrast, no benzodiazepine was prescribed. Our results highlight specific issues that should be addressed in the future to optimize the therapeutic management of neuropathic pain in general practice.

### Knowledge of SFETD recommendations

In our study, one in two GPs declared that they knew the recommendations for neuropathic pain. This proportion is similar to published findings from surveys carried out in general practice to assess knowledge about recommendations for six common diseases. However, the percentage of GPs stating that they applied recommendations (42%) was much higher in our study than reported for other diseases (17%) [35]. This led us to question the veracity of the GPs' statements, particularly because we were unable to detect any impact on the recognition of neuropathic pain and its therapeutic management. The remuneration of participation by a pharmaceutical company with considerable involvement in pain management might have favored complaisant responses. We therefore considered the reported implementation of the recommendations by GPs to be too dubious for a relevant assessment of the impact of recommendations on medical practice. Other methodological approaches, such as the assessment of practices before/after the publication of recommendations, or before/after training, would be more appropriate for such an analysis.

### Strengths and limitations of case-vignettes

Case-vignettes have been used for years to evaluate of the behavior of physicians in the setting of diagnostic testing or treatment decisions. We chose to use this method for our study as it has been shown to be an accurate, valid, feasible and inexpensive tool for measuring the quality of health care [16], [17], [19], [36], [37]. Previous studies have demonstrated the utility of case-vignettes for assessing compliance with recommendations, for measuring physicians' practice performance, particularly for comparisons of different groups of physicians, for identifying deviations from guidelines and physicians with non-ideal approaches to patients due to a lack of knowledge, and for defining areas in which scientific knowledge could be strengthened [19], [38], [39].

This study had several methodological strengths. Case-vignette design was based on epidemiological and descriptive data for neuropathic pain, which we used to create an initial scenario matching current clinical practice in ambulatory care as closely as possible. Case-vignettes were constructed by an expert panel with six members and were further refined after successful testing on a sample of 20 GPs. The assessment criterion was the recognition of neuropathic pain, and the various items proposed in the questionnaire corresponded to the different diagnostic and therapeutic elements addressed in the French recommendations (pain description, clinical examination, first-line medication).

However, our study also had a number of limitations. Men were overrepresented, introducing a selection bias. Nevertheless, the likelihood of recognizing or correctly managing neuropathic pain is unlikely to differ between the sexes, and no significant difference according to sex was identified in this study. This research was sponsored by Pfizer, and we cannot rule out the possibility that this influenced GPs' answers, particularly given the overdiagnosis of neuropathic pain observed.

We selected case-vignettes relating to only four etiologies of chronic pain. The inclusion of a broader range of etiologies would have increased the difficulty of diagnosis and might have generated different results. We presented cases of pure chronic pain that was either neuropathic or nociceptive. The introduction of mixed pain would probably have increased the percentage of misdiagnoses, given the greater complexity of the possible choices.

Despite the great care that we took to ensure that case-vignettes were similar in terms of difficulty (particularly as concerns the number and quality of neuropathic descriptors included in the cases of neuropathic pain), we cannot rule out the possibility that other difficulties influenced the results. For example, case no. 4, “non-neuropathic cancer pain”, which was one of the worst diagnosed cases, had specific features. It was the only case among the four cases of non-neuropathic pain presenting two neuropathic elements. Moreover, these elements appeared ahead of all the other items proposed whereas, in other cases, they were presented at the end of the list of items. These various aspects highlight the difficulties involved in drawing up case-vignettes. Thus, case-vignettes seem to be a tool that is more useful for highlighting weaknesses on which communication efforts should focus, rather than providing figures concerning medical practice. As previously reported, it is not possible with the case-vignette method to ensure that the responses obtain reflect the way that the GPs would behave in everyday patient care, even if the design and wording of the vignettes are kept as close as possible to real conditions [40]. Indeed, this method does not take into account either doctor-patient interactions or the relational aspects involved in real life.

## Conclusions

The complexity of neuropathic chronic pain poses challenges for both management and diagnosis for primary care physicians. Taking into account our findings and the limitations outlined above, this study highlights to poor adoption of SFETD recommendations, with difficulties recognizing neuropathic pain for certain etiologies, insufficient consideration of clinical examination findings, and the paucity of appropriate first-line drugs recalled by GPs. These results may facilitate the design of specific educational programs and interventions aiming to improve the management of neuropathic pain by GPs in France.

## Supporting Information

File S1 (DOCX)
