# Supplementary material for: Adherence of French GPs to Chronic Neuropathic Pain Clinical Guidelines: Results of a Cross-Sectional, Randomized, “e” Case-Vignette Survey
Source: PLoS One. 2014 Apr 18;9(4):e93855. doi: 10.1371/journal.pone.0093855 (PMC3991574; doi:10.1371/journal.pone.0093855)
Supplement: File S1 — (DOCX) [file pone.0093855.s001.docx]

**APPENDIX S1**

**PAIN in diabetic patients**

| Case n°1 Vignette describing a case of painful diabetic polyneuropathy |
| --- |
| Mr T is 68 years old, weighs 95 kg and is 1.75 m tall. The principal elements of his history are non-insulin-dependent diabetes, of 10 years’ duration, and arterial hypertension, which was diagnosed eight years ago. He gave up smoking one year ago. He is currently treated with 1000 mg metformin, 4 mg/day glimepiride and 50 mg/day losartan.  For the last 18 months, he has suffered from bilateral foot and leg pain described as “burning” and “electric shocks” associated with pricking sensations, tingling and numbness. His pain is intense, evaluated at 8/10 on a numerical rating scale. The patient is very anxious and complains of insomnia because contact with bed linen during the night is very painful.  Clinical examination shows no motor deficit, and the Achilles and knee reflexes are present and symmetric. Sensitivity to touch is normal, but the patient displays hyposensitivity to heat, cold and prick stimuli. The peripheral pulses are detectable and there is no detectable murmur in the vascular axes. An EMG carried out one month ago was normal, and a Doppler scan of the leg arteries was also normal. |
| Case n°2 Vignette describing a case of non-neuropathic pain in a cancer patient |
| Mr C is 70 years old, married, and has a history of smoking (15 packets/year), hypercholesterolemia treated with a cholesterol-lowering agent and arterial hypertension treated with angiotensin-converting enzyme inhibitors. He is being managed for colorectal cancer, treated by colectomy followed by platinum salt-based chemotherapy. During chemotherapy, he presented tingling sensations at the extremities of all four limbs.  He complains of pain in both knees, of several months’ duration, with the right side more strongly affected than the left. The pains are deep and dull in nature, and of high intensity (8/10 on a numerical rating scale). They are aggravated by walking or by climbing stairs. During the interview, the patient tells you about his anxiety and his problems sleeping.  Clinical examination reveals genu valgum, with pain on mobilization of the knees. The patient displays hyposensitivity of the feet to touch and prick stimuli. The Achilles reflexes are absent.  X rays show femoral-tibial pinching, which is bilateral, but more pronounced on the right. The patient brings an electromyogram carried out one month previously, which provided evidence of distal peripheral neuropathy of the legs. |

**PAIN in CANCER patients**

| Case n°3 Vignette describing a case of cancer chemotherapy-induced peripheral neuropathy |
| --- |
| Mr C is 70 years old, married, and has a history of smoking (15 packets/year), hypercholesterolemia treated with a cholesterol-lowering agent and arterial hypertension treated with angiotensin-converting enzyme inhibitors. He is being managed for colorectal cancer, treated by colectomy followed by platinum salt-based chemotherapy. During chemotherapy, he presented tingling and pricking sensations, together with pain in the extremities of all four limbs. The pains were described as “burning” and “electric shocks”, predominantly in the lower limbs, aggravated by contact with cold objects. The patient is very anxious and is consulting because he is suffering from pain that keeps him awake, with an intensity rated at 8/10 on a numerical rating scale.  Neurological examination of the feet reveals pain on rubbing and hyposensitivity of the feet to touch and to prick stimuli. Achilles reflexes are absent from both sides. |
| Case n°4 Vignette describing a case of non-neuropathic pain in a diabetic patient |
| Mr S is 72 years old, weighs 95 kg and is 1.75 m tall. He smokes and the principal elements of his history are non-insulin-dependent diabetes of 10 years’ duration, and arterial hypertension diagnosed eight years ago. He is currently treated with 1000 mg metformin, 4 mg/day glimepiride and 50 mg/day losartan. He complains of knee pain of several months’ duration. The pain predominantly affects the right knee. It is deep and dull in nature and is aggravated by walking and climbing stairs. Mr S evaluates the intensity of his pain at 8/10 on a numerical rating scale. During interview, Mr S displays signs of anxiety and reports changes in the quantity and quality of sleep.  Clinical examination shows bilateral genu valgum with painful mobilization of the knees. The osteotendinous reflexes are absent and the patient is insensitive to prick stimuli applied to the soles of the feet. The peripheral pulses are difficult to detect and vascular auscultation reveals a left femoral murmur. Knee X rays show internal femoral-tibial pinching, which is bilateral, but more pronounced on the right. The patient has an electromyogram carried out one month previously, providing evidence of distal peripheral neuropathy of the legs. A Doppler scan of the legs is normal. |

**LOW BACK PAIN**

| Case n°5 Vignette describing a case of lumbo-radiculalgia |
| --- |
| Mr A is 36 years old and works as a care assistant in an operating theatre. He has a history of chronic lower back pain, with several episodes of sciatica leading to an operation on a herniated disk in the right L4 L5 position. He is very anxious. After the operation, the lumbar pain disappeared, but pain persisted in the right leg. This pain is present during rest and is aggravated by exercise and by walking. The patient describes this pain as “burning” and “electric shocks” radiating to the lower part of the thigh, the leg and the back of the right foot. This pain is intense, evaluated at 8/10 on a numerical rating scale, and it keeps the patient awake at night. The patient also describes sensations of numbness and tingling at the back of the thigh. Clinical examination reveals a discrete motor deficit of the lifting of the right foot, but the reflexes are normal. There is mild hyposentivity of the back of the right foot to touch and prick stimuli, although rubbing of this zone immediately triggering pain. |
| Case n°6 Vignette describing a case of non-neuropathic low back pain |
| Mr A, is 55 years old and works as a care assistant in an operating theater. He has a history of non-insulin-dependent diabetes of more than 10 years’ duration, treated by diet and oral antidiabetic drugs. He was alcoholic but gave up drinking two years ago.  He has been suffering from lower back pain radiating to the right buttock for three months, following a trivial fall onto his buttocks. This pain is continuous, intense (8/10 on a numerical rating scale), keeps the patient awake and is aggravated by the slightest movement. The patient describes the pain as being like a dull heaviness on either side of the lumbar vertebrae. He explains that he is very anxious. Clinical examination reveals spinal stiffness and contracture of the paravertebral muscles. Neurological examination shows hyposensitivity of both soles of the feet to prick stimuli. The |

**POST-OPERATIVE PAIN**

| Case n°7 Vignette describing a case of post-operative neuropathic pain |
| --- |
| Mr I is 40 years old. He smokes, weighs 95 kg and is 175 cm tall. The principal element of his history is a complex fracture of the right ankle treated by osteosynthesis one year ago. Mr I has not returned to work as a delivery driver because of persistent pain since his accident.  Pain occurs around the scar when walking and at rest. Pain is described as permanent **burning** associated with a spontaneous **electric shock-like feeling** several times daily. It is an intense pain, rated 8/10 on the numerical rating scale, and keeps the patient awake at night. The patient also experiences strange sensations, such as **pricking** and **tingling**. Mr I is very anxious and he tells you that the most distressing is the pain triggered by the lightest touch, which prevents him from putting his shoes on. He is wearing sandals to the consultation.  Clinical examination shows a right submalleolar scar resulting from surgery with **decreased sensitivity** around the scar (to touch, prick and cold stimuli) and intense **pain triggered merely by light touching** of the periscar area. |
| Case n°8 Vignette describing a case of post-operative non-neuropathic pain |
| Mr I is 40 years old. He smokes, weighs 95 kg and is 175 cm tall. The principal element of his history is a complex fracture of the right ankle treated by osteosynthesis 10 years ago. This fracture rapidly improved on treatment and Mr I was able to return to work as a delivery driver.  He consults his doctor for ankle pain that has been gradually increasing for several months. Pain is more severe at the end of the day and is aggravated by walking or standing for a long time. It is a deep, vice-like, intense pain, rated 8/10 on the numerical rating scale. An interview with the patient reveals anxiety and insomnia.  Clinical examination shows an old right submalleolar scar with a distinct **area of tactile hyposensitivity** around the scar, mild perimalleolar edema, and pain triggered by mobilization of the ankle. |

**Discriminative elements for neuropathic pain** (6 elements in case n°7 *vs* 1 in case n°8).

Non-discriminative elements for neuropathic pain (intensity, comorbidities). Identical in terms of quantity and quality for both cases.

**APPENDIX S2**

**SFETD recommendations on diagnosis and treatment of neuropathic pain in primary care**

**- Key points-**

**DIAGNOSIS**

- Diagnosis of neuropathic pain is based on well*-*conducted patient interview and clinical examination. It is the convergence in elements from clinical history and examination that allows recognizing neuropathic pain.
- Patient's own words should draw attention, some descriptors being more frequently used than others to describe neuropathic pain (burning, electric shock, painful cold)
- Painful symptoms are often associated with abnormal sensations (paresthesia, dysesthesia) which are painless but often unpleasant such as tingling, pins and needles, itching, numbness.
- Clinical examination looks for associated neurological signs (sensory deficits -tactile, thermoreceptive, or nociceptive-) and allows to verify that spontaneous and/or evoked pain is located in an area consistent with a peripheral or central neurological injury.
- DN4 questionnaire is a tool recommended in clinical practice as a valuable aid in the diagnosis of neuropathic pain.


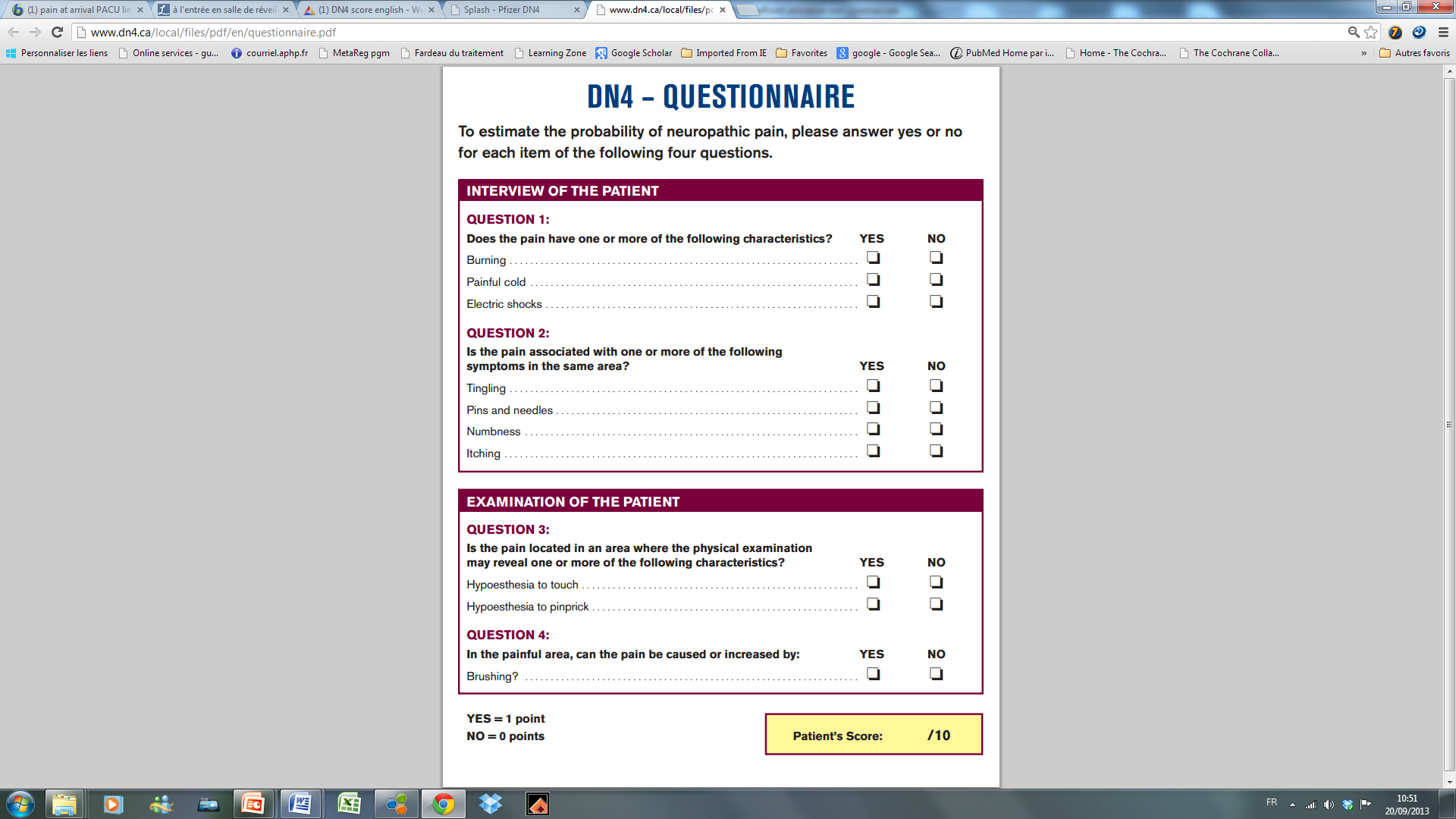


**Therapeutic management**

- Neuropathic pain is not (or hardly) relieved by non-narcotic analgesics such as paracetamol and non-steroidal anti-inflammatory drugs. Other therapeutic classes should be considered.
- A tricyclic antidepressant or an antiepileptic (gabapentin, pregabalin) is recommended as first line monotherapy in neuropathic pain.
- Duloxetine is recommended as first line monotherapy in painful diabetes polyneuropathy.

**APPENDIX S3**

**case-vignette questionnaire**

1. **DIAGNOSIS**

***⮚ In your opinion, which type of pain is the patient most likely to be suffering from?***

- 1. *Neuropathic pain*
  2. *Mechanical pain*
  3. *Inflammatory pain*
  4. *Nociceptive pain*

1. **ELEMENTS GUIDING DIAGNOSIS**

***⮚ Which elements from the clinical history guide diagnosis in this case?***

1. *Description of burning pain, electric shock feeling*
2. *Abnormal sensations: tingling, “pins and needles”*
3. *Insomnia and anxiety*
4. *Pain intensity rated 8 / 10*

***⮚ Which elements from the clinical examination guide diagnosis in this case?***

1. *Pain caused by friction*
2. *Tactile hyposensitivity*
3. *Location at foot level*
4. **THERAPEUTIC STRATEGY**

***⮚ Which first-line analgesic drug(s) could you prescribe?***

1. *Pregabalin*
2. *Ibuprofen*
3. *Amitriptyline*
4. *Acetaminophen/codeine*
5. *Clonazepam*
6. *Gabapentin*
7. *Duloxetine*
8. *Others*
